# Recent Reports of Solid-Phase Cyclohexapeptide Synthesis and Applications

**DOI:** 10.3390/molecules23061475

**Published:** 2018-06-18

**Authors:** Allan M. Prior, Taylor Hori, Ashriel Fishman, Dianqing Sun

**Affiliations:** Department of Pharmaceutical Sciences, The Daniel K. Inouye College of Pharmacy, University of Hawaii at Hilo, 34 Rainbow Drive, Hilo, Hawaii, HI 96720, USA; aprior@hawaii.edu (A.M.P.); th9@hawaii.edu (T.H.); ashriel@hawaii.edu (A.F.)

**Keywords:** cyclohexapeptide, solid-phase synthesis, total synthesis, macrocyclization, macrolactamization, structure–activity relationship, natural products, on-resin cyclization, solution-phase cyclization

## Abstract

Macrocyclic peptides are privileged scaffolds for drug development and constitute a significant portion of macrocyclic drugs on the market today in fields spanning from infectious disease to oncology. Developing orally bioavailable peptide-based drugs remains a challenging task; however, macrocyclization of linear peptides can be an effective strategy to improve membrane permeability, proteolytic stability, oral bioavailability, and overall drug-like characteristics for this class. Significant advances in solid-phase peptide synthesis (SPPS) have enabled the efficient construction of macrocyclic peptide and peptidomimetic libraries with macrolactamization being performed on-resin or in solution phase. The primary goal of this review is to summarize solid-phase cyclohexapeptide synthesis using the on-resin and solution-phase macrocyclization methodologies published since 2013. We also highlight their broad applications ranging from natural product total synthesis, synthetic methodology development, and medicinal chemistry, to drug development and analyses of conformational and physiochemical properties.

## 1. Introduction 

Macrocyclic peptides constitute a significant portion of macrocyclic drugs on the market today and are used in many fields ranging from infectious disease to oncology [1,2]. Recently, reports of antibacterial macrocyclic peptide natural products have demonstrated that macrocyclic peptides are privileged scaffolds for drug development [3]. Historically, the pharmaceutical industry has been cautious with developing macrocyclic drugs because of concerns of higher cost and synthetic challenges associated with lead optimization and scale-up campaigns [1]. Head-to-tail cyclization of linear peptides of three to eight amino acids can be challenging, particularly for peptides containing exclusively the L-configuration [4]. The formation of linear as well as cyclic dimers and oligomers during the cyclization step can compete with head-to-tail cyclization [5]. Additionally, epimerization of the *C*-terminal amino acid is commonly encountered during the *C*-terminal activation step before the cyclization reaction takes place. To date, significant progress has been made in the synthesis of macrocycles, making their efficient construction more feasible [1]. A common strategy to improve head-to-tail cyclization of small peptides involves the incorporation of the “turn-inducing” functionality into a linear peptide sequence such as glycine, proline, pseudoproline, *N*-alkyl, or D-amino acid residue [4,6,7,8]. In the case of the pseudoproline method, a pseudoproline residue, synthesized by the condensation of serine, threonine, or cysteine with an aldehyde or ketone, is incorporated into the peptide sequence pre-cyclization. The pseudoproline residue can then be deprotected post cyclization [6,7]. Coupling reagents in the azabenzotriazole class are the most commonly employed for cyclization of linear peptide precursors, as they give faster rates of cyclization with lower amounts of epimerization, typically less than 10% [4]. Moreover, linear peptides with the D-configuration at their *C*-terminal residue have favorable cyclization kinetics [4]. This may be a result of less steric hindrance during the formation of the peptide bond occurring between the D- and L-amino acids, particularly when bulky side chains are present. Cyclodepsipeptides, with one ester linkage in the macrocyclic backbone [9], have been utilized in the epimerization-free synthesis of cyclopeptides by employing a key *O*-*N*-acyl migration reaction at a serine residue [10,11]. 

Macrocyclic peptides are known to possess some improved membrane permeability [12,13], proteolytic stability [13,14,15,16], oral bioavailability [17,18], and overall drug-like characteristics [13,19,20] over their linear analogues. Moreover, peptide macrocyclization is a way of locking the peptide sequence in a β-strand conformation [15,16], a conformation often recognized by their enzyme targets, such as proteases [15,16,21]. Macrocycles are not completely rigid but still have a degree of flexibility, which facilitates interactions with their receptors [1]. Additionally, the entropic cost of receptor binding may be reduced for macrocyclic drugs compared to their linear equivalents as a result of conformational pre-organization [1]. *N*-Methylation of the cyclic peptide backbone has been shown in some cases to improve peptide metabolic stability [14,22] and oral bioavailability [23] as a result of changes in peptide conformation or steric hindrance [22]. The immunosuppressant drug ciclosporin is a macrocyclic undecapeptide bearing seven *N*-methylated motifs and can be administered as an oral formulation [1,22]. Modification of the cyclohexapeptide backbone to form cyclohexapeptoids can, in some cases, lead to an increase to cell permeability [24]. Most cyclic peptide drugs on the market today are administered parenterally, and only few are orally bioavailable [1], highlighting an ongoing challenge. For this reason, the synthesis and evaluation of novel cyclopeptide scaffolds to expand our understanding of their pharmacokinetic (PK) properties is still at the forefront of many research programs [2,12,23]. 

Solid-phase peptide synthesis (SPPS) was first described in 1963 by Merrifield [25], whereby a growing linear peptide was synthesized in a step-wise fashion while covalently attached to a solid support (resin). In general, excess reagents are used in solid-phase synthesis to help to drive reactions to completion. The solid-supported methodology allows excess reagents to be removed after each step by employing a simple filtration, and the final desired product is obtained after cleavage from the resin [25]. To date, many new advances in SPPS have allowed for the rapid and efficient construction of peptide or peptidomimetic [26] libraries for subsequent biological evaluation and high-throughput screening [27,28]. 

**Figure 1:**
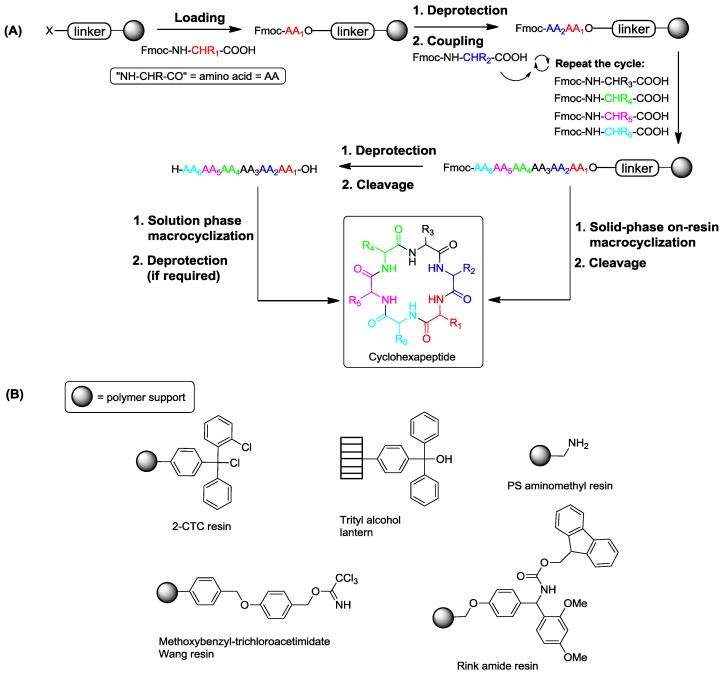
(**A**). Illustration of solid-phase cyclohexapeptide synthesis. (**B**). Resins highlighted in this review.

The current literature base surrounding natural and synthetic macrocyclic peptides is extensive. The structures of these macrocycles have a wide-ranging incorporation of natural and unnatural amino acids as well as different ring sizes. Interestingly, cyclohexapeptides—macrocyclic peptides comprising six amino acids in the ring—are one of the most ubiquitous classes of macrocyclic peptides synthesized by SPPS. Macrocyclic hexapeptides can be obtained directly from on-resin or solution-phase cyclization following the cleavage of a linear hexapeptide precursor from resin (Figure 1). This brief review summarizes the preceding five years’ worth of solid-phase cyclohexapeptide synthesis and its applications, which span from natural product total synthesis, synthetic methodology development, and medicinal chemistry, to drug development and analyses of conformational and physiochemical properties. 

## 2. Solid-Phase Synthesis of Cyclohexapeptides Using Solution-Phase Cyclization

**Figure 2:**
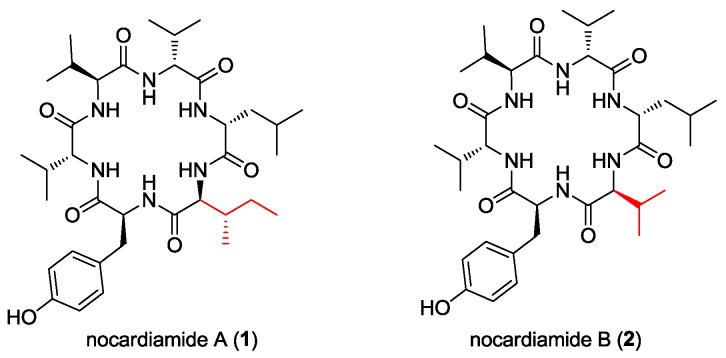
Structures of naturally occurring nocardiamides A (**1**) and B (**2**).

In 2013, Wu et al. reported the structures of two new cyclohexapeptides, nocardiamides A (**1**) and B (**2**), that were isolated from a culture broth of a CNX037 strain of actinomycete, which is a *Nocardiopsis* species (Figure 2) [29]. 

**Scheme 1:**
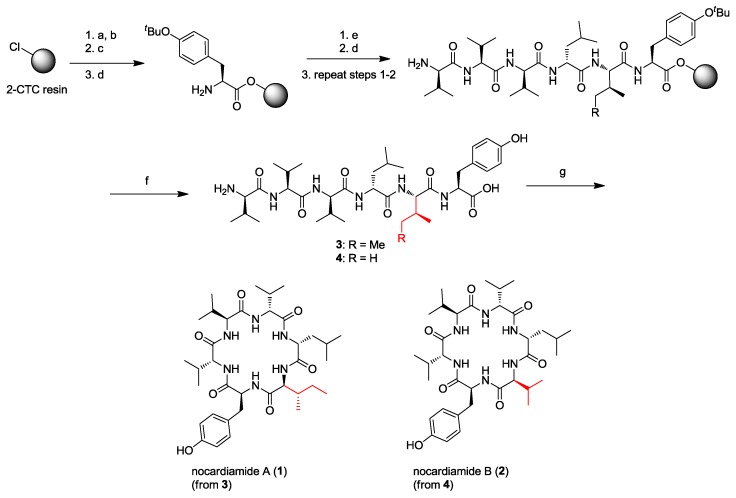
Solid-phase synthesis of nocardiamides A (**1**) and B (**2**) on 2-chlorotrityl chloride (2-CTC) resin. Reagents and conditions: (a) Fmoc-Tyr(^t^Bu)-OH; (b) HBTU, DIPEA, and DMF (r.t., 1.5 h); (c) MeOH (r.t., 0.5 h); (d) 20% piperidine/DMF (r.t., 20 min); (e) Fmoc-AA-OH, HBTU, DIPEA, and DMF (r.t., 0.5 h); (f) TFA/thioanisole/PhOH/1,2-ethanedithiol/H_2_O (82.5/5/5/2.5/5) (r.t.; 31.1% yield for **3** and 47.8% for **4** after RP-HPLC purification); (g) HBTU, DIPEA, and DMF (r.t.; 7.2% yield for nocardiamide A (**1**) and 10.7% for nocardiamide B (**2**) after RP-HPLC purification).

Their structures were confirmed through an independent total synthesis, which helped to confirm the location of the two D- and L-Val residues. The synthesis of **1** and **2** is shown in Scheme 1. Their synthesis used 2-chlorotrityl chloride (2-CTC) resin as a solid support to construct the linear hexapeptides **3** and **4** using classical SPPS, followed by cleavage from the resin using a trifluoroacetic acid TFA-based cleavage cocktail solution. Subsequent *N,N,N′,N′-*tetramethyl-*O*-(1*H*-benzotriazol-1-yl)uronium hexafluorophosphate (HBTU)-mediated solution-phase cyclization of **3** or **4** provided cyclohexapeptides **1** and **2** in 7.2% and 10.7% yields, respectively. Antimicrobial evaluation of **1**–**4** was performed against *Escherichia coli*, *Staphylococcus aureus*, *Enterococcus faecalis*, *Bacillus thuringiensis*, *Bacillus subtilis*, *Micrococcus luteus*, and *Candida albicans*; however, only negligible activity was found. Compounds **1** and **2** were also screened against a human colon carcinoma cell line, HCT-116, and no cell cytotoxicity was observed [29]. 

**Scheme 2:**
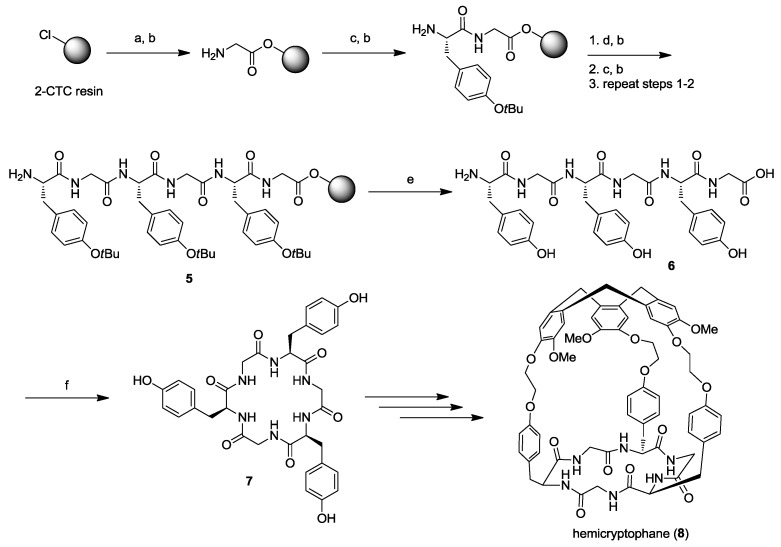
Solid-phase synthesis of cyclohexapeptide **7** on 2-chlorotrityl chloride (2-CTC) resin. Reagents and conditions: (a) 1. Fmoc-Gly-OH, DIPEA, and DCM/DMF (4/1) (r.t., 16 h); 2. MeOH (r.t., 20 min); (b) two treatments with 20% piperidine/DMF (r.t., 5 min); (c) Fmoc-Tyr(^t^Bu)-OH, HATU (0.5M), DIPEA, and DMF (70 °C, 5 min, microwave heating); (d) Fmoc-Gly-OH, HATU (0.5M), DIPEA, and DMF (70 °C, 5 min, microwave heating); (e) three treatments with 5% TFA in CH_3_CN/H_2_O (4:1) (r.t., 20 min, 85%); (f) PyBOP and DMF (r.t., 1 h; quantitative yield after purification by flash chromatography (silica gel)).

In 2013, Cochrane et al. reported the synthesis of the first members of a new class of cyclic-peptide-containing hemicryptophanes (e.g., **8** in Scheme 2) [30]. From this work, the solid-supported linear hexapeptide **5** was prepared from 2-CTC resin by standard microwave-assisted 9-fluorenylmethoxycarbonyl (Fmoc) SPPS (Scheme 2). Cleavage of the hexapeptide from the resin was done using 5% TFA in CH_3_CN/H_2_O (4:1), affording the unprotected linear peptide **6** in 85% yield. Subsequent cyclization of **6** using (benzotriazol-1-yloxy)tripyrrolidinophosphonium hexafluorophosphate (PyBOP) in dimethylformamide (DMF) gave the cyclohexapeptide **7** in quantitative yield after 1 h. Interestingly, cyclization of **6** proceeded much faster than that of its corresponding *O*-tert-butyl protected linear peptide counterpart. Cyclic hexapeptide **7** was used to produce a new class of cyclohexapeptide-containing hemicryptophanes, which were investigated for their enantioselective binding properties by complexation with carnitine [30].

**Scheme 3:**
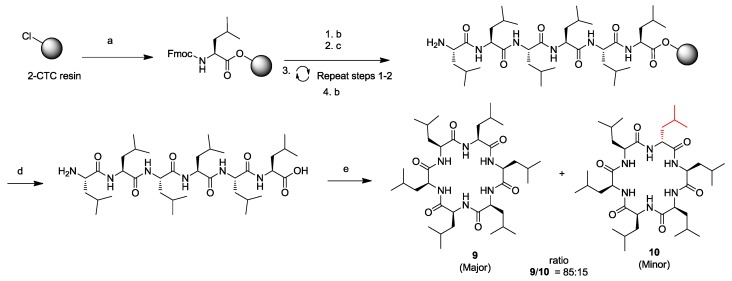
Solid-phase synthesis of **9** and **10** on 2-chlorotrityl chloride (2-CTC) resin. Reagents and conditions: (a) Fmoc-Leu-OH, DIPEA, and DCM (r.t., overnight); (b) treated twice with 50% piperidine/DMF (r.t., 10 min); (c) Fmoc-Leu-OH, HATU, DIPEA, and DMF (r.t., 2 h); (d) TFA/TIPS/H_2_O (95/2.5/2.5) (r.t., 2 h); (e) dropwise addition of linear peptide solution in DMF to solution of PyBOP, DIPEA, and DMF (over 3 h, r.t., overnight; 17% yield for **9** and 3% for **10** after preparative RP-HPLC purification).

Peptide-based therapeutics are notorious for their poor oral bioavailability profiles and low plasma stability, which have limited their use as orally delivered drugs. Although *N*-methylation of cyclohexapeptides was shown to improve oral bioavailability [23], Hill et al. demonstrated that cyclohexaleucine peptides **9** and **10** without *N*-methylation functionality showed some degree of oral bioavailability: 17% and 9%, respectively [12]. Interestingly, the epimer **10** showed ~2-fold lower oral bioavailability than **9** as a result of its notably higher plasma clearance rate (24.1 versus 4.7 mL/min/kg). The reason was suggested to be caused by differences in solvent exposure to the peptide backbone resulting from the conformational change at a Leu residue. Membrane permeability was measured using RRCK and CACO-2 cell monolayers, the former having a lack of active transporters. In the RRCK assay, compounds **9** and **10** showed 2–3-fold greater membrane permeability than the control standard cyclosporin A (CsA). In the CACO-2 assay, **9** and **10** showed similar permeability to CsA (P_app_ ≈ 5 × 10^−6^ cm·s^−1^) [12]. As shown in Scheme 3, the cyclohexaleucine peptide **9** was synthesized using SPPS on 2-CTC resin as a linear hexapeptide precursor, followed by cleavage from resin and solution-phase cyclization under dilute conditions. Epimer **10** was formed during the final cyclization step but could be successfully separated out during purification using reverse-phase high-performance liquid chromatography (RP-HPLC). The observed epimerization was due to a well-known epimerization process that takes place during the carboxylic acid activation step prior to cyclization [31,32]. In this example, the epimer ratio of **9** and **10** was 85:15 [12].

**Figure 3:**
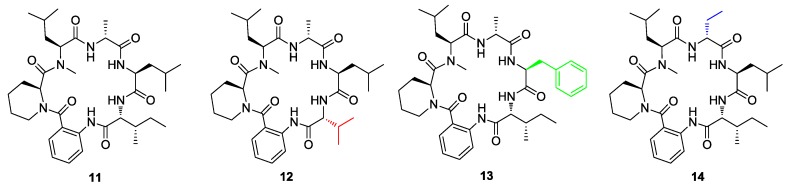
Structures of naturally occurring cyclohexapeptides **11**–**14**.

**Scheme 4:**
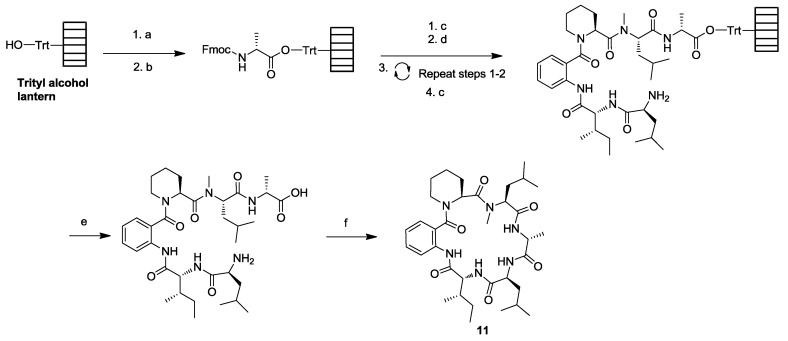
Solid-phase peptide synthesis of **11** on trityl alcohol lantern. Reagents and conditions: (a) AcCl and DCM (r.t., 4 h); (b) Fmoc-D-Ala-OH, DIPEA, and DCM (r.t., 12 h); (c) 20% piperidine/DMF (r.t., 1 h); (d) Fmoc-AA-OH, DIC, HOBt, and DMF (r.t., 12 h); (e) 30% hexafluoroisopropanol (HFIP)/DCM (r.t., 1 h); (f) HATU, DIPEA, and DCM (r.t., 3 h); yields (over 13 steps): 29% (**11**), 41% (**12**), 39% (**13**), and 29% (**14**)).

In 2014, Masuda et al. reported the total synthesis and insecticidal activity of the cyclohexapeptide natural products PF1171A (**11**), C (**12**), F (**13**), and G (**14**) (Figure 3) [33]. The producing organisms are commonly fungi, such as *Hamigera avellanea* [34], *Acremonium* [35] or *Penicillium* [36] species. Of particular interest is the high degree of D-amino acid incorporation (D-Ala, D-Aba, D-allo-Ile, and D-Val) within the macrocyclic scaffold. These natural products also contain non-proteinogenic anthranilic acid (Ant) and L-pipecolinic acid (Pip) residues, suggesting that a non-ribosomal biosynthetic pathway may have been used by the producing organisms. The enhanced bioavailability of these natural products can be attributed to improved cell permeability (via passive diffusion) and higher metabolic stability due to their cyclic nature and incorporation of D-amino acids [33]. The synthesis of **11**–**14** was achieved via the initial construction of linear hexapeptides by SPPS using a trityl alcohol SynPhase Lantern solid support. A representative synthesis for the construction of cyclohexapeptide **11** is shown in Scheme 4. The SPPS started by attaching Fmoc-D-Ala-OH for **11**–**13** or Fmoc-D-Aba-OH for **14**, as this allowed the final solution-phase HBTU-mediated macrocyclization step to proceed with the least amount of steric hindrance [33]. The *N*-methyl-Leu, L-pipecolinic acid, and anthranilic acid functionality seen in **11**-**14** was incorporated into the peptide sequence employing Fmoc-L-MeLeu-OH, Fmoc-L-Pip-OH or Fmoc-Ant-OH during SPPS, respectively. When coupling to the weakly nucleophilic amino function of the N-terminal anthranilic acid residue, the Fmoc-amino acid chloride of D-allo-Ile or D-Val was generated in situ using triphosgene prior to coupling [33]. The linear hexapeptides were cleaved from the resin using 30% hexafluoroisopropanol (HFIP) in dichloromethane (DCM) and cyclized in solution using 1-[bis(dimethylamino)methylene]-1*H*-1,2,3-triazolo[4,5-*b*]pyridinium 3-oxide hexafluorophosphate (HATU) and *N*,*N*-diisopropylethylamine (DIPEA) in DCM at room temperature for 3 h to afford **11**–**14**. The natural products **11**–**14** were tested in a fourth-instar larvae assay and were all found to have paralytic activity against silkworm larvae. The stereochemistry of the D-Ala side chain was found to be crucial for paralytic activity, as side-chain epimers of **11** and **12** having L-Ala in place of D-Ala showed little activity. 

**Figure 4:**
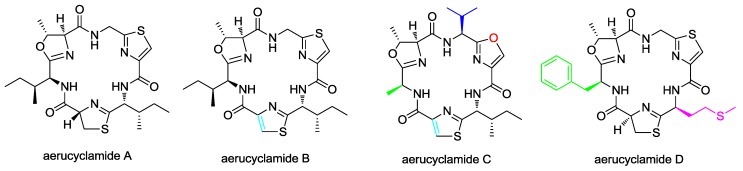
Structures of aerucyclamides A–D.

**Scheme 5:**
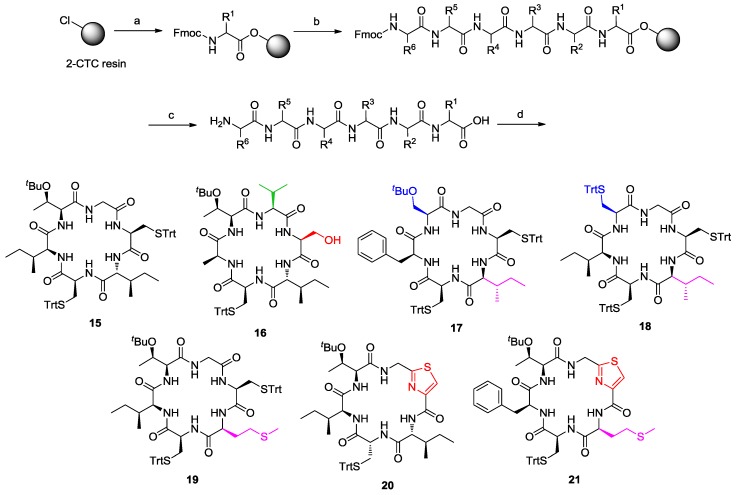
Solid-phase peptide synthesis of **15**–**21** on 2-chlorotrityl chloride (2-CTC) resin. Reagents and conditions: (a) Fmoc-AA-OH (or Fmoc-Thz-OH in the synthesis of **20** and **21**), DIPEA, and DCM (r.t., 1 h, capped with MeOH, r.t., 0.5 h); (b) 1. deprotection: 20% piperidine/DMF (r.t., 2 × 5 min followed by 1 × 10 min); 2. coupling: Fmoc-AA-OH, HOAt, DIC, and DMF (r.t., 2 h); 3. steps 1–2 repeated; (c) cleavage: four treatments with 1% TFA/DCM (r.t., 3 min); (d) macrocyclization: (concentration of 1–5 mM) HBTU, DIPEA, DMAP, and DCM (r.t., 3–5 days; cyclization yields: 59% (**15**), 55% (**16**), 48% (**17**), 66% (**18**), 54% (**19**), 83% (**20**), and 40% (**21**)).

In 2014, Peña et al. reported the synthesis and antimalarial and antitrypanosomal activity of seven novel cyclohexapeptides **15**–**21** with **20** and **21** incorporating interesting thiazole functionality [37]. The study was prompted by earlier reports of cyanobacterium *Microcystis aeruginosa* PCC 7806 natural products, aerucyclamides A–D (Figure 4), which displayed promising micromolar IC_50_ (50% of maximal inhibitory concentration) values against a K1 chloroquine-resistant strain of *Plasmodium falciparum* [38,39]. The SPPS of linear hexapeptide precursors was conducted using 2-CTC resin and standard Fmoc chemistry (Scheme 5). The first Fmoc-amino acid was loaded onto 2-CTC resin in DCM in the presence of DIPEA, followed by the capping of unreacted resin sites with methanol. For the synthesis of **20** and **21**, the 2-CTC resin was first loaded with an Fmoc-protected thiazole residue (Fmoc-Thz-OH) using analogous conditions. Subsequent amino acids were installed via iterative deprotection (20% piperidine in DMF) and coupling (*N,N'*-diisopropylcarbodiimide (DIC) and 3-hydroxytriazolo[4,5-*b*]pyridine (HOAt) in DMF) steps. After cleavage of linear hexapeptides from the resin using 1% TFA in DCM, macrocyclizations were performed using HBTU, DIPEA, and catalytic 4-dimethylaminopyridine (DMAP) in DCM under dilute conditions (1–5 mM). The coupling site for macrocyclization was chosen to occur at *N*-terminal glycine for **15**, *C*-terminal glycine for **17**–**19**, or *C*-terminal thiazole for **20**–**21**, as this provided the least degree of steric hindrance, resulting in favorable yields [37]. Moreover, cyclization of linear precursors to afford **17**–**21** took place at a *C*-terminal glycine or thiazole to prevent epimerization.

Cyclohexapeptides **15**–**21** were screened with *P. falciparum* K1 and infective *T. b. brucei* assays. Of particular interest was the promising antimalarial activity shown by **17**, **20**, and **21** against *P. falciparum* K1, with EC_50_ (50% of maximal effective concentration) values of 0.19, 0.19, and 0.41 µM, respectively, and no observed cell cytotoxicity against murine macrophages [37]. Also noteworthy was the antitrypanosomal activity of **15** and **19**–**21** against *T. b. brucei*, with EC_50_ values of 1.06, 2.1, 3.0, and 2.8 µM, respectively [37].

**Figure 5:**
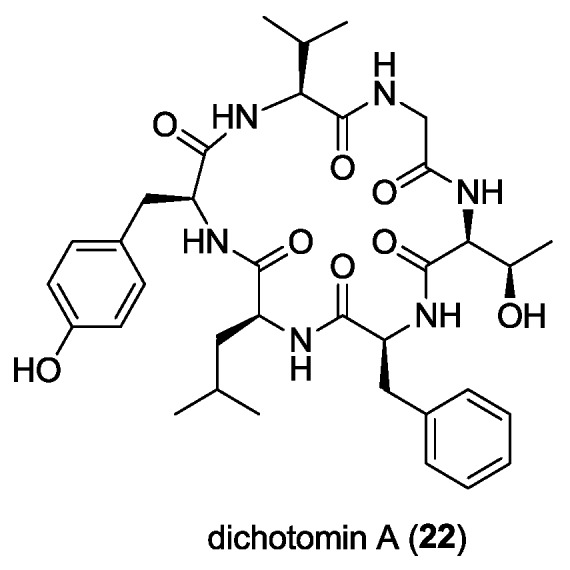
Structure of dichotomin A (**22**).

**Scheme 6:**
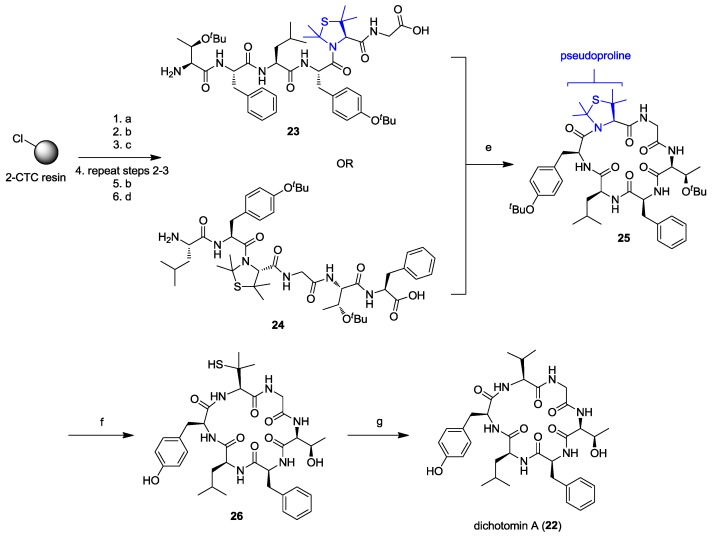
Solid-phase peptide synthesis of dichotomin A (**22**) on 2-chlorotrityl chloride (2-CTC) resin. Reagents and conditions: (a) 1. Fmoc-Gly-OH (for **23**) or Fmoc-Phe-OH (for **24**), DIPEA, and DCM; 2. DCM/MeOH/DIPEA (17:2:1); (b) 10% piperidine/DMF; (c) Fmoc-AA-OH, HBTU, DIPEA, and NMP; (d) 20% HFIP/DCM; (e) (concentration of 1 mM) DMTMM·BF_4_, DIPEA, and DMF (r.t., 3 h; yield of **25**: 84% from **23** and 87% from **24**)); (f) TFMSA/water (2:1) (r.t., 4 h, 33%); (g) NiCl_2_, NaBH_4_, and MeOH (0 °C, 0.5 h, 24%).

In 2015, Wong et al. reported the total synthesis of dichotomin A (**22**) (Figure 5) from linear peptide precursors **23** and **24** that contain penicillamine-derived pseudoproline residue (Scheme 6) [8]. The utilization of a pseudoproline residue in place of the proteinogenic Val residue during the synthesis of dichotomin A was a protecting group stratergy and a way to improve head-to-tail cyclization of linear peptide precursors because of the ability of pseudoproline to induce a turn or “kink” in the peptide backbone as well as to aid peptide solubility [8]. The pseudoproline residue was deprotected and converted into the desired Val residue post cyclization to afford dichotomin A (**22**). Dichotomin A was prepared from two different disconnection sites within the macrocyclic ring (Scheme 6). The first linear peptide precursor **23** contained a non-epimerizable *C*-terminal glycine and an *N*-terminal *O*-tert-butyl protected threonine residue. The second peptide precursor **24** contained an epimerizable *N*-terminal leucine and a *C*-terminal phenylalanine, creating a more sterically hindered cyclization site and providing a way to test the robustness of this methodology [8]. Linear peptides **23** and **24** containing pseudoproline residues were synthesized on 2-CTC resin using standard Fmoc-strategy SPPS with HBTU as the coupling reagent and 10% piperidine in DMF for Fmoc deprotection. Cleavage from resin was done using HFIP in DCM while keeping the side-chain protecting groups intact. The conformations of **23** and **24** were established using **^1^**H nuclear magnetic resonance (NMR) spectroscopy and rotating-frame Overhauser effect spectroscopy (ROESY) experiments, which also confirmed a single set of resonance structures for each peptide. Macrocycle **25** was obtained from linear peptides **23** or **24** containing a pseudoproline residue in less than 3 h and in good to excellent yields (78–88%) using 4-(4,6-dimethoxy-1,3,5-triazin-2-yl)-4-methylmorpholinium tetrafluoroborate (DMTMM·BF_4_). In contrast, cyclization of analogous linear peptide precursors containing a native Val residue had much slower reaction kinetics, requiring 3 days for reaction completion and with lower yields (33–36%). The pseudoproline and *O*-tert-butyl-Thr residues in **25** were simultaneously deprotected using trifluoromethanesulfonic acid (TFMSA)/water (2:1, *v*/*v*) to give **26** followed by desulfurization using NiCl_2_ and NaBH_4_ in MeOH to afford dichotomin A (**22**) in 24% yield. 

**Figure 6:**
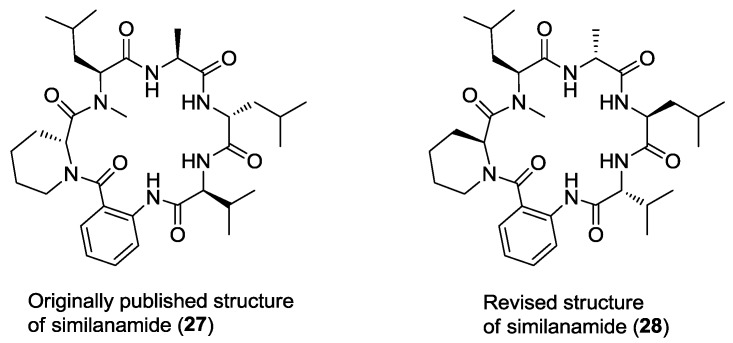
Structures of cyclohexapeptides **27** and **28**.

In 2015, Prompanya et al. isolated a cyclohexapeptide from a culture of the marine sponge-associated fungus *Aspergillus similanensis* KUFA 0013 and named it similanamide (**27**) (Figure 6) [40]. Masuda et al. undertook the total synthesis of similanamide (**27**) in 2015 [41] but noticed discrepancies when comparing the ^1^H and ^13^C-NMR data of their synthesized similanamide (**27**) with reported data [40]. The reported data for similanamide (**27**) did, however, agree favorably with a previously synthesized diastereomer **28** [41]. This led to the structural revision of similanamide (**27**) to the structurally similar diastereomer **28** in 2015 [41].

**Figure 7:**
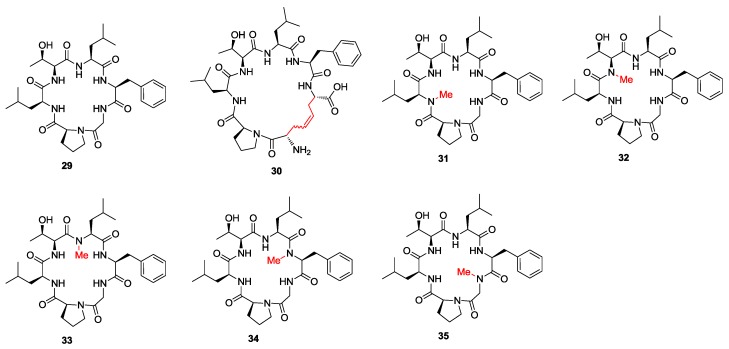
Structures of dianthin G (**29**), “dicarba” analogue **30**, and *N*-methyl analogues **31**–**35**.

In 2016, Amso et al. reported the synthesis of cyclohexapeptide dianthin G (cyclo-Pro-Leu-Thr-Leu-Phe-Gly, **29**) as well as nine of its analogues, including *N*^α^-methylated derivatives (**31**–**35**) and conformationally constrained cyclic “dicarba” bridged analogues (e.g., **30**) (Figure 7) [42,43]. The in vitro osteoblast proliferation activity of the synthesized compounds was determined. Dianthin G (**29**) and its *N*^α^-methylated derivatives (**31**–**35**) were synthesized by SPPS followed by cleavage and solution-phase head-to-tail macrolactamization. 

**Scheme 7:**
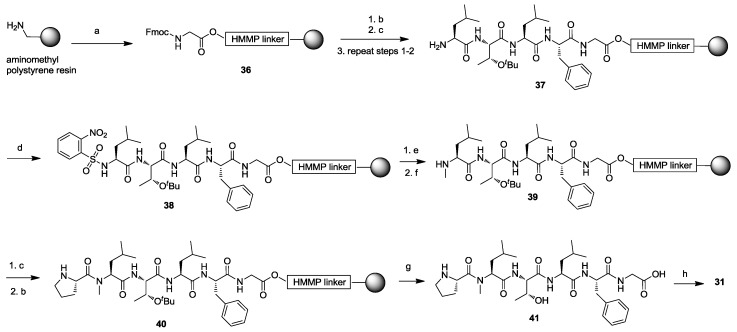
Representative solid-phase peptide synthesis of **31** on aminomethyl polystyrene resin. Reagents and conditions: (a) Fmoc-Gly-O-HMMP-OH, DIC, DCM, and DMF (r.t., 4 h); (b) deprotection: 20% piperidine/DMF (r.t., 2 × 5 min); (c) coupling: Fmoc-AA-OH, HATU, DIPEA, and DMF (r.t., 45 min); (d) 2-nitrobenzenesulfonyl chloride, sym-collidine, and NMP (r.t., 2 × 15 min); (e) dimethyl sulfate, DBU, and NMP (r.t., 2 × 5 min); (f) 2-mercaptoethanol, DBU, and NMP (r.t., 2 × 5 min); (g) TFA/TIPS/H_2_O (95/2.5/2.5) (r.t., 3 h); (h) HBTU, 6-Cl-HOBt, DIPEA, DCM, and DMF (r.t., 36 h; overall yields: 52% (**29**), 38% (**31**), 22% (**32**), 51% (**33**), 43% (**34**), 39% (**35**)).

The SPPS of native cyclohexapeptide dianthin G (**29**) was reported previously [43] but was later adapted for the synthesis of methylated analogues **31**–**34** (Scheme 7). The *N*^α^-methylated derivatives **31**–**34** were synthesized from aminomethyl polystyrene resin and the 3-(4-hydroxymethylphenoxy)propionic acid) (HMPP) linker; however, the synthesis of **35** required the use of a more hindered 2-CTC resin to prevent the formation of undesired diketopiperazine by-products [42]. A representative synthesis of cyclohexapeptide **31** is shown in Scheme 7. The aminomethyl polystyrene resin was first loaded with Fmoc-Gly-HMPP-OH to form **36**. The solid-supported linear pentapeptide **37** was constructed on-resin using classical SPPS. The terminal amino function was activated by reacting with 2-nitrobenzenesulfonyl chloride to form **38**, followed by methylation and deprotection to afford **39**. After attaching the final proline residue, hexapeptide **40** was cleaved from the resin to form **41**, which was cyclized using HBTU-mediated solution-phase cyclization to afford **31**. 

Cyclic dicarba analogues contained a non-native dicarba bridge, for example, **30**, and were synthesized by on-resin Grubbs’ ring-closing metathesis (RCM) and then cleaved from resin to form **30** as an inseparable mixture of cis and trans isomers [42]. An *N*^α^-methyl amide bond scan of the synthesized dianthin G, which was done to investigate the effect that altering amide bonds had on osteoblast proliferation, found that all native peptide bonds contained in the primary sequence of dianthin G (**29**) were of importance for osteoblast proliferation activity. From in vitro studies, native dianthin G (**29**) and a dicarba bridged analogue (at 10^−8^ M) were found to increase the numbers of human osteoblasts without having a significant effect on osteoclast differentiation or development. An inseparable Z/E mixture of olefins in a 2:1 ratio with a β-sheet-like secondary structure similar to that of native dianthin G (**29**) was determined through spectroscopic analysis of the dicarba analogue. It was suggested that this secondary structure is important for the bone activity associated with dianthin peptides.

**Scheme 8:**
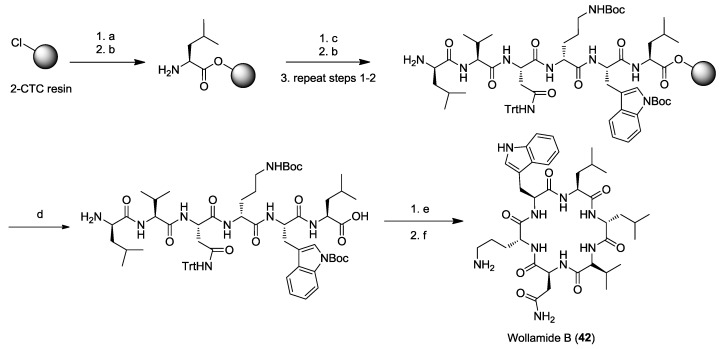
Solid-phase peptide synthesis of **42** on 2-chlorotrityl chloride (2-CTC) resin. Reagents and conditions: (a) Fmoc-Leu-OH, DIPEA, and DCM (r.t., 2 h); (b) 20% piperidine/DMF (r.t., 2 × 10 min); (c) Fmoc-AA-OH, HBTU, DIPEA, and NMP (r.t., 1 h); (d) 20% HFIP/DCM (r.t., 1 h); (e) (concentration of 1 mM) HATU, HOBt, DIPEA, and DMF (0 °C to r.t.; r.t., 3 days; cyclization yield: 68%); (f) TFA/TIPS/H_2_O (95/2.5/2.5) (r.t., 3 h; yield of **42**: 91%).

In 2017, Asfaw et al. reported the synthesis of cyclohexapeptide wollamide B (**42**) and 24 of its analogues [44]. The Fmoc-based SPPS of a linear hexapeptide precursor was followed by solution-phase macrocyclization and cleavage of protecting groups, as described in Scheme 8. The first amino acid (Fmoc-Leu-OH) was loaded onto 2-CTC resin by using DIPEA/DCM followed by capping with methanol. HATU and DIPEA in NMP were used during the coupling steps to elongate the peptides. The *N*-terminal Fmoc group was deprotected after each round of coupling using 20% piperidine in DMF. The resin was treated with 20% HFIP in DCM to complete cleavage of the linear hexapeptide precursors. Macrocyclization was done using HATU, hydroxybenzotriazole (HOBt), and DIPEA in DMF to produce a crude cyclic hexapeptide that was then purified via column chromatography. Lastly, the Trt and Boc side-chain protecting groups were removed using a TFA/triisopropylsilane (TIPS)/H_2_O solution (95/2.5/2.5) to afford wollamide B (**47**) in 91% yield after purification by silica gel chromatography. 

**Figure 8:**
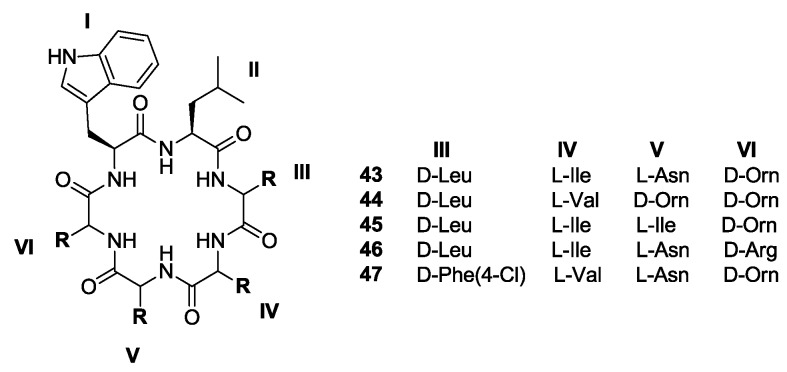
Structures of cyclohexapeptides **43**–**47**.

The antimycobacterial activities as well as the in vitro drug metabolism and PK (absorption, distribution, metabolism, and excretion—ADME) profiles of **42** and structural analogues were investigated. Wollamide B (**42**) was found to have notable aqueous solubility, moderate affinity for plasma protein albumin, modest lipophilicity, poor passive permeability through artificial membranes, significant in vitro plasma stability, high microsomal metabolic stability, and no toxicity in HepG2 cells for concentrations of up to 50 µM. Notably, five of the synthesized wollamide B analogues (**43**–**47**; Figure 8) displayed potent antimycobacterial activity (minimum inhibitory concentration (MIC) of ≤3.1 µM) and no toxicity in HepG2 cells for concentrations of up to 100 µM. Compounds **43** and **46** also showed an optimal balance between antimycobacterial activity and PK properties. Overall, the synthesized wollamides displayed notable plasma stability and aqueous solubility with moderate to low metabolic stability.

**Figure 9:**
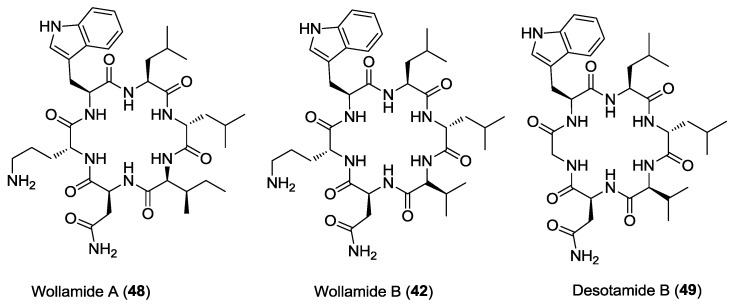
Wollamides A (**48**), and B (**42**) and desotamide B (**49**).

**Scheme 9:**
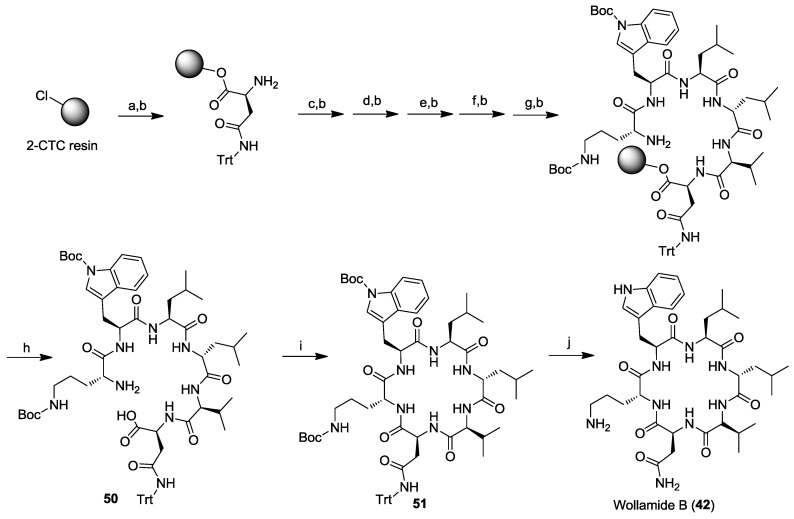
Solid-phase peptide synthesis of **42** on 2-chlorotrityl chloride (2-CTC) resin. Reagents and conditions: (a) Fmoc-Asn(Trt)-OH, DIPEA, and DCM (r.t., 3 h); (b) 25% 4-methylpiperidine/DMF; (c) Fmoc-Val-OH, DIC, HOBt, and DMF/DCM (1:1) (r.t., 4 h); (d) Fmoc-D-Leu-OH, DIC, HOBt, and DMF/DCM (1:1) (r.t., 4 h); (e) Fmoc-Leu-OH, DIC, HOBt, and DMF/DCM (1:1) (r.t., 4 h); (f) Fmoc-Trp(Boc)-OH, DIC, HOBt, and DMF/DCM (1:1) (r.t., 4 h); (g) Fmoc-D-Orn(Boc)-OH, DIC, HOBt, and DMF/DCM (1:1) (r.t., 4 h); (h) HFIP/DCM (1:4) (r.t., 0.5 h); (i) (concentration of 1 mM) HBTU, DIPEA, and DMF (r.t., 0.5 h; cyclization yield of **51**: 72%); (**j**) TFA/TIPS/DCM (50:5:45) (r.t., 0.5 h, 42%).

In 2017, our group reported the total synthesis of the natural products wollamides A (**48**) and B (**42**) and desotamide B (**49**) (Figure 9) using SPPS of linear hexapeptide precursors followed by cleavage and solution-phase cyclization [45]. A representative synthesis that was used to access wollamide B (**42**) is highlighted in Scheme 9. The first Fmoc amino acid (Fmoc-Asn(Trt)-OH) was loaded onto 2-CTC resin with the aid of DIPEA in DCM. The hexapeptide sequence corresponding to wollamide B (D-Orn-Trp-Leu-D-Leu-Val-Asn) was synthesized on-resin via repeated coupling and deprotection steps, as shown in Scheme 9. Cleavage of the linear hexapeptide from the resin using the mild cleavage reagent HFIP resulted in **50** having side-chain residue protecting groups still attached. The solution-phase macrocyclization was done using HBTU and DIPEA in DMF, providing the protected cyclohexapeptide **51**. Wollamide B (**42**) was obtained after removal of the side-chain protecting groups of **51** and final purification using flash column chromatography on silica gel. An optimization study investigated the efficiency of the macrocyclization step occurring at each of the six peptide bond sites. This determined that macrocyclization of the linear hexapeptide precursor between terminal L-Leu and D-Leu residues provided the most efficient macrocyclization without any detectable epimerization [45]. 

**Figure 10:**
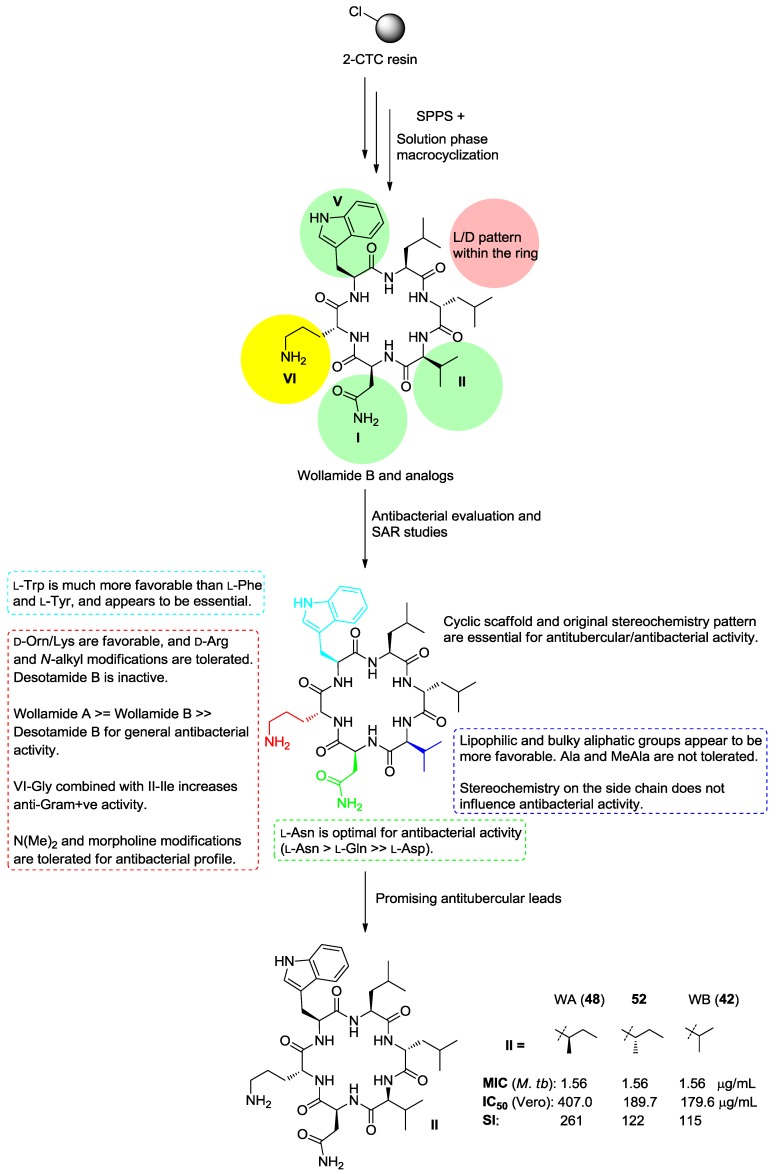
Synthesis and structure–activity relationship (SAR) studies of cyclohexapeptides **48**, **52**, and **42**.

In 2018, our group reported the antitubercular and antibacterial activities of wollamides A (**48**) and B (**42**) and desotamide B (**49**) as well as structural analogues thereof [46]. The optimized synthetic route to access these compounds was described previously [45]. The 27 peptides’ library was screened against a panel of Gram-positive and -negative bacterial pathogens, which discovered that wollamides A (**48**) and B (**42**) and the position-II L-Ile analogue **52** exhibited promising antibacterial activity against *Mycobacterium tuberculosis*, with MIC values of 1.56 µg/mL and favorable selectivity indexes (SIs) of >100 (Figure 10) [46]. The cyclic nature of the wollamide cyclohexapeptides was crucial for their antibacterial activity, as the corresponding linear hexapeptide precursor did not show activity even at the highest concentration tested (200 µg/mL) [46]. The residues at positions II and VI were found to have a major impact on the activity and selectivity, and hence further structure–activity relationship (SAR) studies that focus on optimizing these residues are highly warranted.

## 3. Solid-Phase Cyclohexapeptide Synthesis Using On-Resin Cyclization

**Figure 11:**
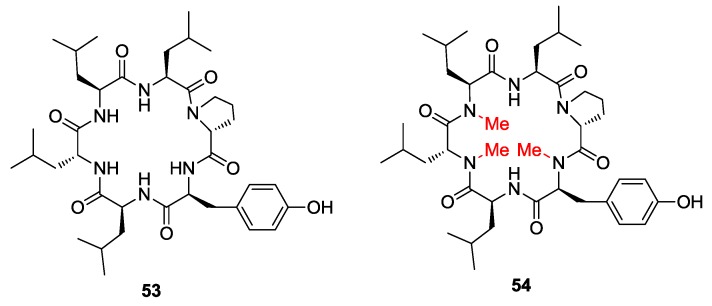
Structures of cyclohexapeptides **53** and **54**.

**Scheme 10:**
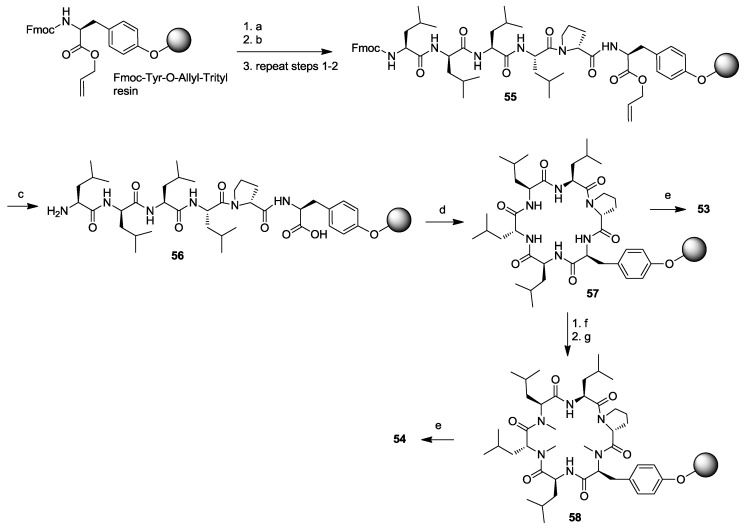
Solid-phase synthesis of **53** and **54** on trityl resin. Reagents and conditions: (a) 20% 4-methylpiperidine/DMF (r.t., 2 × 5 min); (b) Fmoc-AA-OH, PyOxim, DIPEA, and DMF/NMP (1:1) (r.t., 45 min); (c) Pd(PPh_3_)_4_ and 10% piperidine/THF (r.t., 2 h); (d) HATU, HOBt, DIPEA, and DMF/NMP (1:1) (r.t., 18 h); (e) 10% TFA/DCM (r.t., 4 × 5 min); (f) LiO*^t^*Bu and THF (r.t., 0.5 h); (g) MeI and DMSO (r.t., 0.5 h).

In 2015, Lewis et al. synthesized two cyclic hexapeptides, **53** and **54** (Figure 11), in order to probe their cell permeability and PK properties, including oral bioavailability [23]. They demonstrated that the tri-*N*-methylated peptide **54** experienced increased cell permeability, higher plasma protein binding, and decreased clearance rates compared to the non-methylated variant **53** [23]. This resulted in a favorable bioavailability of 30% for the tri-*N*-methylated cyclohexapeptide **54**. The cyclic hexapeptides cyclo-Leu-D-Leu–Leu-Leu-D-Pro-Tyr (**53**) and cyclo-Leu-NMe-D-Leu-NMe-Leu-Leu-D-Pro-NMe-Tyr (**54**) were synthesized using traditional SPPS starting from a trityl resin preloaded with allyl ester Fmoc-Tyr, which was resin-linked via the Tyr side-chain hydroxyl group (Scheme 10). The resin-linked linear hexapeptide **55** was constructed using a sequence of coupling and deprotection steps. The allyl and *N*-terminal Fmoc groups were removed using Pd(PPh_3_)_4_ and 10% piperidine/THF, respectively, to give **56**, which was cyclized on-resin using HATU/HOBt to give the resin-attached cyclohexapeptide **57**. Cleavage of **57** from the resin using TFA provided **53**. Cyclohexapeptide **54** was synthesized from **57** via the global and selective introduction of *N*-methyl groups using a LiO^t^Bu base, followed by MeI to provide resin-attached trimethylated derivative **58** (Scheme 10). Cleavage of **58** from the resin afforded **54**. An alternative synthesis of **54** was also investigated and involved a stepwise construction of the trimethylated linear hexapeptide using traditional SPPS followed by cleavage and solution-phase macrocyclization. Although two routes for the synthesis of **54** were investigated, the first route involving global *N*-methylation of the resin-bound cyclohexapeptide **57** followed by cleavage was more efficient and provided crude **54** in higher purity before final purification. 

In 2015, Wodtke et al. reported the design and synthesis of cyclohexapeptide analogues **59**–**61** containing an amino acid sequence inspired by the Asp-Glu-Lys-Ser (DEKS) motif of *N*-terminal telopeptide of type I collagen [47]. It was suggested that the DEKS motif adopts a β-turn conformation upon docking with its receptor; in addition, the β-turn conformation of the DEKS motif can be stabilized by incorporating it into a cyclized peptide backbone along with strategic introduction of D-Pro and Lys(4-fluorobenzoyl) residues [47]. The presence of the 4-fluorobenzoyl group in the second Lys of **59**–**61** could have an application as a radiolabeling site, if required, by incorporating fluorine-18 [47]. The cyclohexapeptide **59** was resistant to bovine trypsin-mediated degradation over 30 min, during which time its corresponding linear analogue was completely digested. This demonstrated how cyclized peptides offer enhanced metabolic stability over their linear counterparts [47]. 

**Scheme 11:**
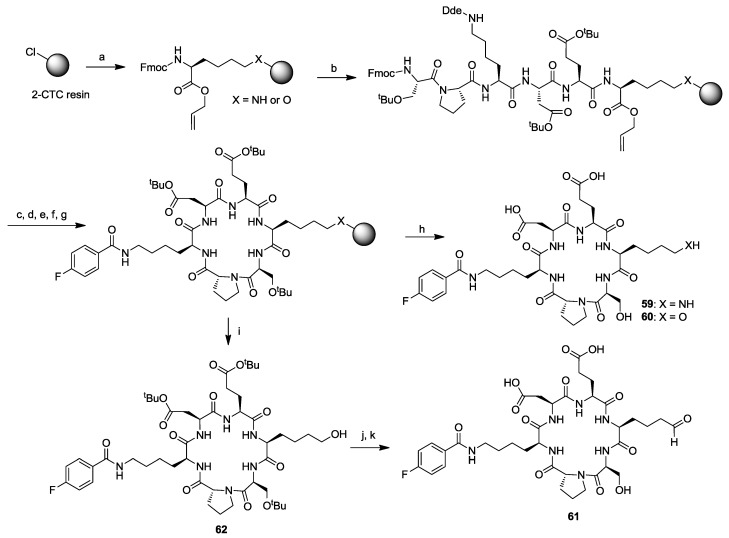
Solid-phase peptide synthesis of **59–62** on 2-chlorotrityl chloride (2-CTC) resin. Reagents and conditions: (a) 1. (for X=NH) Fmoc-Lys-OAll, DIPEA, and THF (r.t., 2 h); 2. DCM/MeOH/DIPEA (17:1:2) (r.t., 3 × 2 min); 1. (for X=O), Fmoc-Hln-OAll, pyridine, and DCM/DMF (1:1) (r.t., 64 h); 2. DCM/MeOH/DIPEA (17:1:2) (r.t., 3 × 2 min); (b) 1. deprotection: 20% piperidine, 0.1M HOBt, and DMF (microwave irradiation: 35 W, 75 °C, 30 s, followed by 44 W, 75 °C, 3 min); 2. coupling: Fmoc-AA-OH, HBTU, DIPEA, and DMF (microwave irradiation: 21 W, 75 °C, 5 min); 3. steps 1–2 repeated; (c) Pd(PPh_3_)_4_, and DCM/NMM/acetic acid (8:2:1) (r.t., 4 h); (d) 20% piperidine/DMF (r.t., 2 × 8 min); (e) HATU, DIPEA, and DMF (r.t., 4 h); (f) 2% N_2_H_4_ and DMF (r.t., 5–12 × 5 min); (g) 4-fluorobenzoyl chloride, NEt_3_, and DCM (r.t., 2 h); (h) TFA/TES/H_2_O (95/2.5/2.5) (r.t., 3 h); (i) TFA/TES/DCM (1:5:94) (r.t., 0.5 h); (j) Dess–Martin periodinane and DCM (r.t., 3 h); (k) TFA/DCM (9:1) (r.t., 1 h; overall yields: 80% (**59**), 30% (**60**), and 25% (**61**)).

The synthesis of cyclohexapeptide **59**–**61** was carried out according to Scheme 11. Firstly, Fmoc-Lys-OAll or Fmoc-Hnl-OAll was loaded onto 2-CTC resin (attached via side chain) in THF and DIPEA followed by capping unreacted resin sites with methanol. The subsequent Fmoc-amino acids were added to the sequence using standard microwave-assisted SPPS, 20% piperidine, and 0.1M HOBt in DMF for the Fmoc deprotection steps and Fmoc-amino acid, HBTU, and DIPEA in NMP for the coupling steps. The resin-bound linear hexapeptide was cyclized on-resin using HATU and DIPEA in DMF after deprotection of the *C*-terminal allyl and *N*-terminal Fmoc groups with Pd(PPh_3_)_4_ and 20% piperidine in DMF, respectively. The 4-fluorobenzoyl moiety was installed onto the lysine side chain using 4-fluorobenzoyl chloride after firstly removing the 1-(4,4-dimethyl-2,6-dioxocyclohex-1-ylidene)-3-ethyl (Dde) protecting group with hydrazine. The cyclohexapeptide was cleaved from the resin with simultaneous removal of the side-chain protecting groups using a cleavage cocktail solution comprising TFA/triethylsilane (TES) (95/2.5/2.5) to give **59**–**60** in good yields. Compound **61** was synthesized by cleaving the cyclohexapeptide **62** from the resin using mild acid conditions for TFA/TES/DCM (1:5:94) to give **62** with side-chain protecting groups attached. Finally, Dess–Martin periodinane oxidation of **62** followed by removal of the side-chain protecting groups provided **61** in good yield. 

**Scheme 12:**
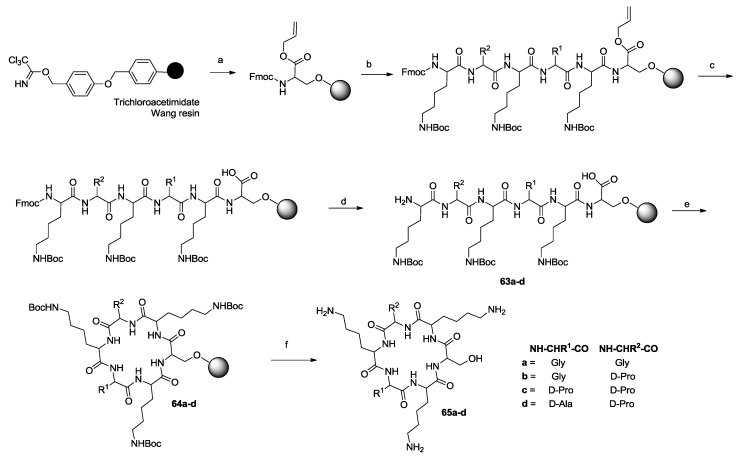
Solid-phase peptide synthesis (SPPS) of cyclohexapeptides **65a**–**d**. Reagents and conditions: (a) Fmoc-D-Ser-OAll, BF_3_·OEt_2_, and dry TFA (r.t., 1 h); (b) SPPS for five cycles: deprotection: 20% piperidine/DMF; coupling: Fmoc-AA-OH, BOP, HOBt, DIPEA, and DMF; (c) Pd(PPh_3_)_4_ and PhSiH_3_; (d) 20% piperidine/DMF; (e) PyBOP, HOBt, DIPEA, and DMF (r.t., 17 h); (f) 95% TFA (r.t., 2 h; yields: 15% (**65a**), 38% (**65b**), 63% (**65c**), and 13% (**65d**).

In 2016, Jikyo reported the synthesis of cyclic hexapeptides **65a**–**d** using an on-resin head-to-tail cyclization strategy on trichloroacetimidate Wang resin (Scheme 12) [48]. The D-Ser side chain of Fmoc-D-Ser-OAll was anchored to the trichloroacetimidate Wang resin using BF_3_·OEt_2_ in dry TFA. Iterative coupling and deprotection steps using BOP/HOBt/DIPEA and 20% piperidine/DMF, respectively, for five cycles constructed resin-bound linear hexapeptide intermediates having Fmoc/Boc/OAll protecting groups intact. Pd(PPh_3_)_4_ in CHCl_3_/AcOH/*N*-methylmorpholine (NMM) was used under anhydrous conditions to complete *C-*terminal *O*-allyl deprotection before the addition of 20% piperidine in DMF to remove *N*-terminal Fmoc, which afforded anchored linear peptides **63a**–**d** having deprotected *C*- and *N-*terminals. On-resin cyclization to afford **64a**–**d** was achieved with PyBOP/HOBt/DIPEA as the coupling agent, and cleavage from the resin using 95% TFA resulted in cyclohexapeptides **65a**–**d** in 13–63% yields. Interestingly, on-resin cyclization of **64c**, having two D-Pro residues in the chain, gave the highest yield of cyclized product **65c** with a yield of 63%. 

**Scheme 13:**
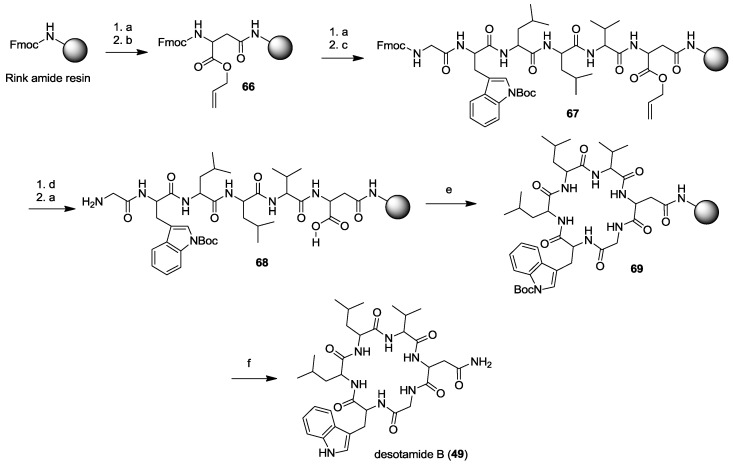
Representative solid-phase peptide synthesis (SPPS) of **49** on Rink amide resin. Reagents and conditions: (a) 20% piperidine/DMF (r.t., 5 × 10 min); (b) Fmoc-Asp-OAll, HCTU, DIPEA, and DMF (r.t., 1 h); (c) SPPS: coupling: Fmoc-AA-OH, HCTU, DIPEA, and DMF (r.t., 1 h); deprotection: 20% piperidine/DMF (r.t.; 5 × 10 min); coupling/deprotection steps repeated. (d) Pd(PPh_3_)_4_, phenylsilane, and DCM; (e) PyAOP, HOAt, NMM, and NMP (r.t., 12 h); (f) TFA/H_2_O/phenol/TIPS (88:5:5:2, *v/v/v/v*) (r.t., 2 h).

In 2018, Chen et al. reported the synthesis and antibacterial evaluation of cyclohexapeptides desotamide B (**49**) and wollamide B (**42**) in addition to a series of their structural analogues by utilizing an on-resin head-to-tail cyclization strategy (Scheme 13) [49]. Fmoc-Asp-OAll was first anchored to the Rink Amide AM resin through the use of the coupling reagent *O*-(1H-6-chlorobenzotriazole-1-yl)-1,1,3,3-tetramethyluronium hexafluorophosphate (HCTU)/DIPEA to form the resin-bond amino acid **66**. SPPS was used to form the linear peptide **67**, which contained different Fmoc protected amino acids. The allyl group was uncapped with Pd(PPh_3_)_4_ and phenylsilane. The *N*-terminal amino group was then released by using a treatment of 20% piperidine/DMF to form the linear peptide precursor **68**. The on-resin cyclization step included treatment with (7-azabenzotriazol-1-yloxy)tripyrrolidinophosphonium hexafluorophosphate (PyAOP)/HOAt/NMM in NMP for 12 h to produce the protected cyclohexapeptide **69** on-resin. The target cyclohexapeptides were produced after final cleavage and global deprotection with TFA/phenol/water/TIPS (88:5:5:2, *v*/*v*/*v*/*v*). 

**Figure 12:**
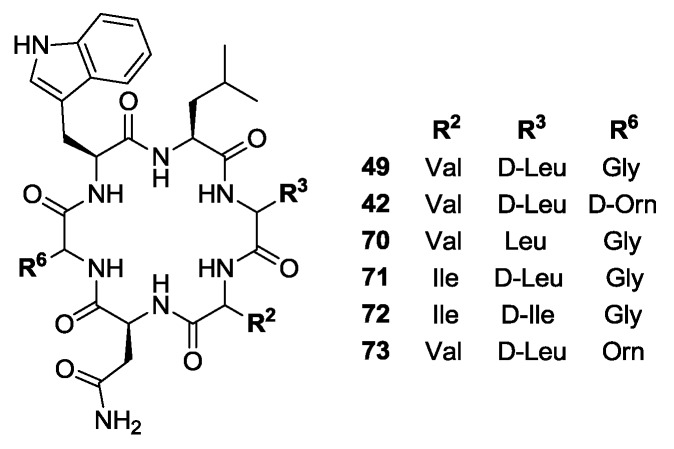
Structures of desotamide B (**49**), wollamide B (**42**), and cyclohexapeptide analogues **70**–**73**.

Cyclohexapeptide **71** (Figure 12) showed inhibitory antibacterial activity for methicillin-resistant *S. aureus* (MRSA)2 (MIC = 128 µg/mL), MRSA4 (MIC = 32 µg/mL), MRSA5 (MIC = 64 µg/mL), and *S. aureus* (MIC = 64 µg/mL), which was about 2-fold increase in activity compared to desotamide B (**49**). It was therefore suggested that replacing Val with Ile improves bioactivity. Compounds **70** and **72** showed a loss of activity (MIC > 128 µg/mL), suggesting that D-Leu is necessary for antibacterial activity. The loss of activity of **73** suggested that D-Orn may be required for the antibacterial activity of wollamide B (**42**). However, it was also suggested that D-Orn may increase cytotoxicity, as wollamide B (**42**) showed higher cytotoxicity than **73** in MCF-7 and HepG-2 assays**.** Nearly all of the synthesized cyclopeptides lacked cytotoxicity (IC_50_ > 100 µM) against both human tumor cells MCF-7 and HepG-2, except for wollamide B (**42**), which exhibited cytotoxicity against HepG-2 (IC_50_ = 79.2 µM).

**Figure 13:**
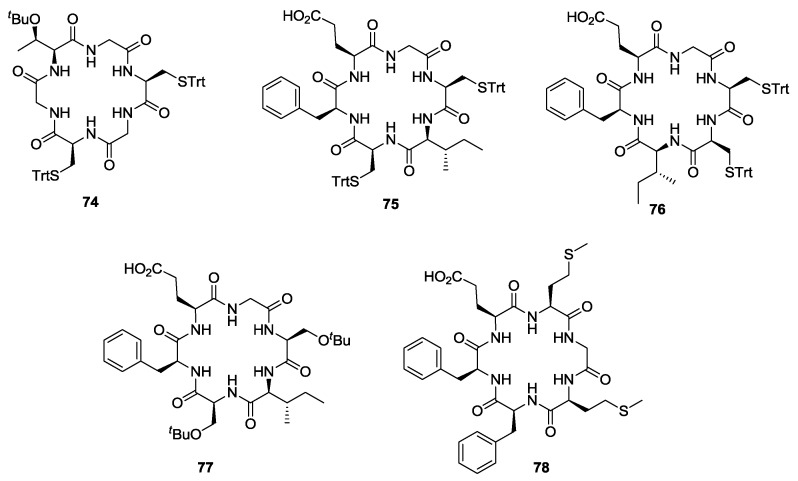
Structures of cyclohexapeptides **74**–**78**.

**Scheme 14:**
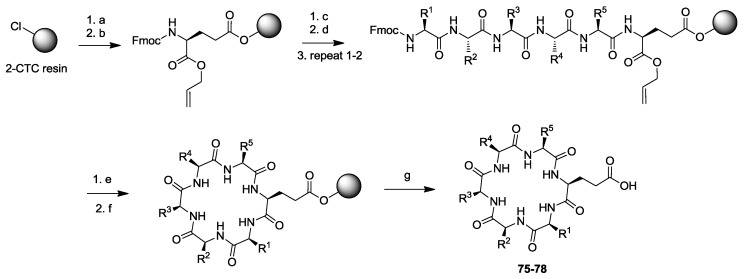
Solid-phase peptide synthesis of **75**–**78** on 2-chlorotrityl chloride (2-CTC) resin. Reagents and conditions: (a) Fmoc-Glu-OAll, DIPEA, and DMF; (b) capped with MeOH; (c) deprotection: 20% piperidine/DMF; (d) coupling: Fmoc-AA-OH, HBTU, DIPEA, and DMF (r.t., 1–2 h); (e) Pd(PPh_3_)_4_ and 10% piperidine/THF (r.t., 3 h); (f) DIC, Cl-HOBt, and DMF/DCM (8:2) (r.t., overnight); (g) 1% TFA/DCM (r.t., 2–3 min; overall yield: 51% (**76**), 63% (**77**), 46% (**78**), and 80% (**79**)).

In 2018, Fagundez et al. synthesized cyclohexapeptides via Fmoc/SPPS by using 2-CTC resin followed by macrolactamization either on-resin (**75**–**78**) (Figure 13) or in solution phase after cleavage of linear peptide precursors from resin [50]. A general method for the on-resin cyclization of cyclohexapeptides **75**–**78** is shown in Scheme 14. The synthesis of **74** consists of the peptide sequence being synthesized on the resin in a similar fashion as is discussed above and being cleaved before undergoing macrocyclization in the solution phase. The compounds produced through on-resin cyclization allowed new derivatives to be synthesized as a result of the presence of a free carboxylic acid. Solution-phase cyclization resulted in the ability to produce more diverse cyclic peptides; however, the on-resin route was more convenient, as larger amounts of product with higher yield and acceptable purity could be produced.

The activity against the chloroquine-resistant K1 strain of *P. falciparum* for each of the synthesized cyclopeptides was determined. Two cyclopentapeptides, which were also synthesized and compared to the cyclic hexapeptides, were found to have less activity. Six of the synthesized cyclic hexapeptides exhibited sub-micromolar activity against *P. falciparum* K1. Hexapeptides **75** and **76** exhibited a free carboxylic group from Glu, which permitted the production of derivatives or products with more soluble salts. Cyclic hexapeptide **74** cyclo-Cys(Trt)-Gly-Thr(^t^Bu)-Gly-Cys(Trt)-Gly was determined to be very active as well as selective against *P. falciparum* (EC_50_ = 28 nM). It was suggested that the biological activity of **74** was affected by substitution of the larger hydrophobic amino acids Phe, Met, and Ile contained in **17** and **19** by Gly, as well as by the retention of one Thr and two Cys.

Together, in the on-resin head-to-tail cyclization strategy, the peptide stays anchored to the resin throughout the final cyclization step, whereas in classical methods reported in previous studies, cleavage of the on-resin linear precursors occurred before cyclization in the solution phase. It was suggested that the on-resin head-to-tail cyclization strategy could improve the process of synthesizing cyclic peptides as well as reduce the quantity of product lost during synthesis.

## 4. Conclusions

In conclusion, cyclohexapeptides represent an important class of natural products and medicinal molecules. Compared to their linear hexapeptide counterparts, macrocyclic hexapeptides often possess improved cell permeability, higher metabolic stability, and enhanced bioavailability as a result of their cyclic nature and incorporation of unnatural amino acids. SPPS has enabled the rapid, efficient synthesis of hexapeptide and peptidomimetic libraries for subsequent biological evaluation and high-throughput screening. In this review, we summarize recent methods used in the successful construction of cyclohexapeptides using SPPS followed by on-resin or solution-phase cyclization. We also highlight recent advances in solid-phase hexapeptide synthesis and their applications, ranging from natural product total synthesis, synthetic methodology development, medicinal activities, and drug development, to analyses of conformational and physiochemical properties. 

Head-to-tail cyclization of linear peptides can be accomplished by either on-resin or solution-phase macrolactamization once they are released from the resin. The advantage of on-resin cyclization is that the formation of linear and/or cyclic oligomeric side products can be minimized. In addition, cyclization on-resin can shorten the synthetic route and minimize purification steps, although the reaction progress is more challenging to monitor. In the case of solution-phase cyclization, performing the reaction under dilute conditions (1–5 mM) can favor cyclization over dimer/oligomer formation. In some examples, adding the linear peptide precursor in a dropwise fashion to maintain high dilution was used successfully. Utilizing a turn-inducing pseudoproline residue can greatly enhance cyclization rates, although it is required to be deprotected post cyclization, with additional reaction step(s). On the other hand, epimerization of the *C*-terminal amino acid during cyclization can be attenuated by employing efficient coupling reagents, such as the azabenzotriazole class. Furthermore, epimerization can be completely avoided by choosing to cyclize at a non-epimerizable residue such as *C*-terminal Gly (when applicable) or by employing the method of *O*-*N*-acyl migration on cyclodepsipeptides precursors. Among various solid supports, 2-CTC resin remains one of the most popular resins in SPPS because of its mild acidolytic cleavage conditions as well as steric effect, preventing diketopiperazine formation. 

## Abbreviations

The following abbreviations are used in this manuscript:

ADME	Absorption, distribution, metabolism and excretion
BOP	(Benzotriazol-1-yloxy)tris(dimethylamino)phosphonium hexafluorophosphate
CsA	Cyclosporin A
2-CTC	2-Chlorotrityl chloride
DBU	1,8-Diazabicyclo[5.4.0]undec-7-ene
DCM	Dichloromethane
Dde	1-(4,4-Dimethyl-2,6-dioxocyclohex-1-ylidene)-3-ethyl
DIC	*N,N′*-Diisopropylcarbodiimide
DIPEA	*N,N*-Diisopropylethylamine
DMAP	4-Dimethylaminopyridine
DMF	Dimethylformamide
DMTMM.BF_4_	4-(4,6-Dimethoxy-1,3,5-triazin-2-yl)-4-methylmorpholinium tetrafluoroborate
EC_50_	50% of Maximal effective concentration
Fmoc	9-Fluorenylmethoxycarbonyl
Fmoc-AA-OH	Fmoc protected amino acid
HATU	1-[Bis(dimethylamino)methylene]-1*H*-1,2,3-triazolo[4,5-*b*]pyridinium 3-oxide hexafluorophosphate
HBTU	*N,N,N′,N′*-Tetramethyl-*O-*(1*H*-benzotriazol-1-yl)uronium hexafluorophosphate
HCTU	*O*-(1*H*-6-Chlorobenzotriazole-1-yl)-1,1,3,3-tetramethyluronium hexafluorophosphate
HFIP	1,1,1,3,3,3-Hexafluoro-2-propanol
HMPP	3-(4-Hydroxymethylphenoxy)propionic acid
HOAt	3-Hydroxytriazolo[4,5-*b*]pyridine
HOBt	Hydroxybenzotriazole
IC_50_	50% of Maximal inhibitory concentration
MIC	Minimum inhibitory concentration
MRSA	Methicillin-resistant *S. aureus*
NMM	*N*-Methylmorpholine
NMP	*N*-Methyl-2-pyrrolidone
NMR	Nuclear magnetic resonance
PK	Pharmacokinetic
PyAOP	(7-Azabenzotriazol-1-yloxy)tripyrrolidinophosphonium hexafluorophosphate
PyBOP	(Benzotriazol-1-yloxy)tripyrrolidinophosphonium hexafluorophosphate
PyOxim	[Ethyl cyano(hydroxyimino)acetato-*O*^2^]tri-1-pyrrolidinylphosphonium hexafluorophosphate
RP-HPLC	Reverse phase-high-performance liquid chromatography
r.t.	Room temperature
SAR	Structure-activity relationship
SI	Selectivity index
SPPS	Solid-phase peptide synthesis
TES	Triethylsilane
TFA	Trifluoroacetic acid
TFMSA	Trifluoromethanesulfonic acid
THF	Tetrahydrofuran
TIPS	Triisopropylsilane
WA	Wollamide A
WB	Wollamide B
